# Learning to manage complexity through simulation: students’ challenges and possible strategies

**DOI:** 10.1007/s40037-016-0275-3

**Published:** 2016-05-31

**Authors:** Gerard J. Gormley, Tara Fenwick

**Affiliations:** School of Medicine, Dentistry and Biomedical Sciences, Centre for Medical Education, Queen’s University Belfast, BT7 1NN Belfast, Northern Ireland, UK; School of Education, University of Stirling, Stirling, Scotland, UK

**Keywords:** Complexity, Simulation, Medical students

## Abstract

Many have called for medical students to learn how to manage complexity in healthcare. This study examines the nuances of students’ challenges in coping with a complex simulation learning activity, using concepts from complexity theory, and suggests strategies to help them better understand and manage complexity.

Wearing video glasses, participants took part in a simulation ward-based exercise that incorporated characteristics of complexity. Video footage was used to elicit interviews, which were transcribed. Using complexity theory as a theoretical lens, an iterative approach was taken to identify the challenges that participants faced and possible coping strategies using both interview transcripts and video footage.

Students’ challenges in coping with clinical complexity included being: a) unprepared for ‘diving in’, b) caught in an escalating system, c) captured by the patient, and d) unable to assert boundaries of acceptable practice.

Many characteristics of complexity can be recreated in a ward-based simulation learning activity, affording learners an embodied and immersive experience of these complexity challenges. Possible strategies for managing complexity themes include: a) taking time to size up the system, b) attuning to what emerges, c) reducing complexity, d) boundary practices, and e) working with uncertainty. This study signals pedagogical opportunities for recognizing and dealing with complexity.

## What this paper adds

- Healthcare professionals often manage complexity in daily clinical practice. Many have called for medical students to learn how to manage complexity in healthcare. Complexity theory has the potential both to help learners and educators understand how complexity works and develop strategies for managing it. In this study, we use complexity theory to examine students’ challenges in coping with simulation learning, and suggest strategies to help them better manage complexity. Many characteristics of complexity can be recreated in a simulation learning activity, affording learners an embodied experience of these complexity challenges and signalling potential pedagogical strategies for managing complexity.

## Introduction

In considering futures of medical education, many such as Bleakley and Mennin have called into question the emphasis on protocol and certainty, and argued that students must learn to manage effectively the dynamic complexity of multi-faceted clinical situations [1, 2]. Towards this end, some have pointed to the utility of ideas from *complexity theory* both to help medical students and educators understand how complexity works and to develop strategies for managing it [3, 4]. In other areas of professional education, complexity theory has been widely applied to understanding learning processes and to improve pedagogies [5–8]. Specifically for simulation education in the healthcare professions, some researchers show how *sociomaterial theories* such as complexity can open up the design of simulation to better emulate clinical settings, and to maximize the possibilities of simulation for student learning [9–12]. These authors call for more research and practice using complexity theory to help understand and improve learning in simulation. This article contributes to this line of enquiry, reporting a study of simulation education that incorporated complexity concepts.

## Understanding complexity theory

Complexity theory concepts have been well rehearsed elsewhere [1–4], so we will provide only a brief introduction here. For some decades now, educators have been using these concepts to explain learning dynamics [6–8]. Emergence, for example, is the phenomenon of unpredictable patterns produced through a myriad of interactions that continuously generate new possibilities and adaptations. Proximity of the system’s elements helps to ensure continuing interactions, while diversity among these elements helps ensure that interactions remain dynamic. Fluctuating changes to the system’s state, often called ‘pertubation’ in complexity terms, emerge through these dynamic interactions to stimulate new possibilities. In most clinical settings this occurs naturally as different demands, issues and unexpected situations converge. These disordering dynamics are always held in tension with ordering limitations. Self-organization (a process where the overall patterns of the system arise out of local interactions between smaller components of the system) is a characteristic of complex systems such as a brain, a dialogue, or a creative meeting, where there is no external force imposing essential principles of organizing. This characteristic permits novel possibilities to emerge, as well as problematic patterns such as extreme oscillations, escalations of reaction-counter-reaction, obsessive loops, etc. Multiple feedback loops, both positive and negative, act to either amplify and escalate or counteract the emerging patterns. Overall indeterminacy is the result, such that linear trajectories cannot be predicted, and no one individual or agent can see or understand the whole system.

These sorts of concepts help to understand not only learning processes, but also everyday clinical situations in organizational work. For the analysis conducted in this study, we chose four concepts in particular to work with: *emergence, fluctuations, feedback loops*, and the *socio-material relations* that produce action, discussed further in the analysis section. These concepts are often used in complexity-informed discussions of professional practice [2, 3, 8]. Actors (elements which have the capacity to act) in complex systems continually adapt to the interactions around them, often experimenting with new possibilities as well as being steered by the emerging system dynamics and self-organizing patterns. In highly responsive complex systems, actors are sensitively attuned to the dynamics around them. They notice, sense, feel, hear and respond to small fluctuations. They participate with other elements in a process of ‘co-specification’ whereby their actions and intents are closely interconnected and mutually, almost intuitively, shaped along with those around them. Complexity analysts point to examples in the natural world, such as the swarming phenomena of flocking birds, to illustrate how these dynamics of close continual attention (often called ‘attunement’ in complexity terms) and response might be productively imitated in human systems. A key point is that the individuals and elements that interrelate and work together in a complex system are not only the humans, but also the non-human materials: objects, bodies, settings, technologies and texts. These social and material dynamics are not just co-present, but also entangled with one another in ways that generate emergent possibilities and patterns.

## Complex ward-based simulation

Medical simulation cannot accurately be described as a genuinely complex adaptive system, as others have argued [10]. It is, to a greater or lesser extent depending on the design, technologies, and openness of the scenarios, manufactured. While students encounter unexpected difficulty and the stress of performance in simulation, they know well that ultimately both they and the patients are safe regardless of their actions. The situations are constituted according to pedagogical rather than clinical purposes: students know that the primary objective is the practice and assessment of their own learning rather than patient care.

At Queen’s University Belfast (QUB) an inter-professional simulation ward-based activity was developed by a team of health professionals, academics, psychologists, drama educators, simulation technologists and medical students as part of a teaching activity. The aim was to create a highly immersive teaching environment that would allow senior medical students to have a reactive and experimental learning experience, without the risk of harming patients. We aimed to promote characteristics of complexity into the design of the ward-based simulation activity including: *emergence, proximity and diversity, perturbation, and self-organization*. The veracity of the ward-based simulation depended not only on the explicit cues at the focus of the scenario, but also on the implicit multisensory (e. g. playing an audio background recording of a busy clinical ward), emotional (e. g. an anxious patient), environmental material (e. g. background medical equipment and health profession uniforms), psychological (e. g. cognitively loading with multiple tasks) cues [13].

Crucially, using clinical manikins risks dehumanizing the patient-learner relationship in simulation-based learning. Therefore we worked with real simulated patients and instructed them using activities designed to allow them to be comfortable with the content and characterization needed for the scenarios. Improvisation theatre techniques were also used to facilitate their development of emergent and complex interactions with learners. For the purposes of this study the overall scenario was framed in the typical ward experience and duties of a junior doctor; however, relatively complex ethical dilemmas *(e. g. end of life issues or a colleague displaying unprofessional practice) *emerged during the course of the scenario. We aimed for students to recognize the evolving ethical dilemma and begin to manage the situation in the dynamic and acute setting of a (simulated) ward. The scenario had an inter-professional context with nursing staff and administrators present on the ward. Ultimately we wanted to afford learners an opportunity to gain a deeper understanding of the complex decision-making processes and behaviours experienced by practising healthcare professionals, based on the principles of complexity theory.

This study aimed to gain a deep understanding of students’ challenges in coping with the complexity they encountered in such a high fidelity inter-professional simulation environment. In this article we highlight key difficulties experienced by students that hampered their ability to perform competently, and strategies for managing complexity suggested by these difficulties that might be taught explicitly to medical students.

## Methods

### Setting and context

The study was carried out in the Queen’s University Belfast, where the medical degree programme follows a five year undergraduate curricular model. Students have clinical exposure and simulation-based learning activities throughout curriculum.

## Recruitment and sampling

A maximal variation sampling was used to recruit participants for this study in order to purposively sample for heterogeneity. Fourth year medical students (in academic year 2013–2014) were invited by email to participate in the study. Students in the fourth year of their studies will have had some prior experience of ward-based simulation learning activities, but this will be the first time they will have encountered an ethical dilemma in the context of simulation. Sampling aimed to strike a balance between the deep understanding of participants’ experiences and the broader insights gained by sampling a larger number of participants. Hence sample sizes are generally smaller, allowing more thoughtful analysis and not being overwhelmed by the volume of data. Therefore we aimed to investigate eight participant experiences in the simulation scenarios. A matrix of willing participants and their demographic characteristics (i. e. gender, age and ethnicity) was used to select a maximal variation sample. Informed written consent was obtained from participants. Ethical approval was obtained from the Schools Research Ethics Committee in advance of the study (Ref: 13/36v2).

## Living simulated ward activity

The ward-based simulated teaching activity described earlier in this paper was used for the purposes of this study. Two participants at a time took part in the simulated ward activity (in different parts of the simulation ward). Each participant was asked to consider the activity as if this were their first day as a ‘junior doctor’ working in a clinical ward. The overall ward-based scenario was planned to last up to a maximum of 15 minutes. Participants were asked to carry out a range of clinical duties (separately) but were unaware that an ethical dilemma would emerge separately for each of the participants (namely: family conflict regarding an end of life case and a breach of confidentiality involving the unprofessional behaviour displayed by a colleague). Ultimately we aimed for participants to recognize and initially begin to manage an emerging ethical dilemma. Scripted simulated patients and relatives were present and interacted with the ‘junior doctors’ in the scenario. Inter-professionally, scripted nursing staff and administrators were also present and interacted with the ‘junior doctors’ throughout the scenario.

## Data capture

**Fig. 1:**
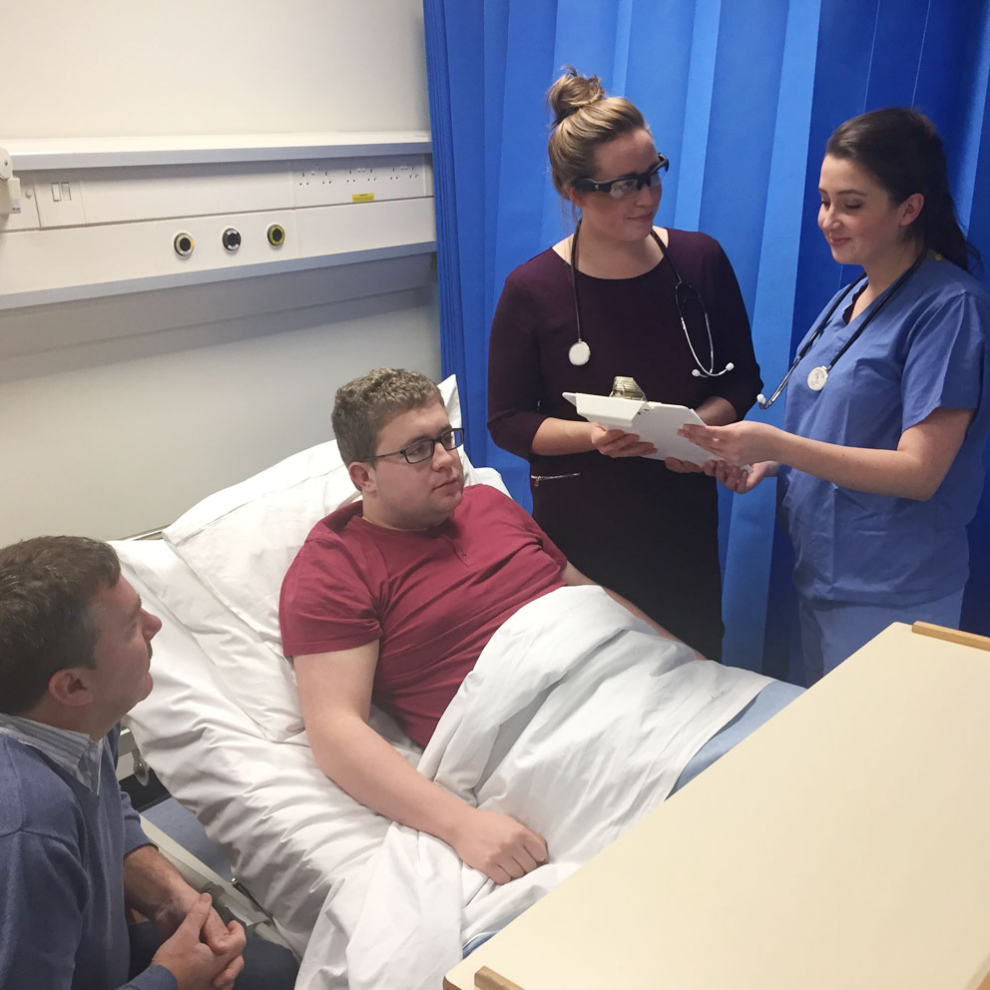
Illustration of simulation ward activity with medical student wearing digital video glasses

Participants wore unobtrusive video glasses (SunnyCam™ HD) to capture, in a personal perspective video, footage of their simulated ward-based experience (Fig. 1). Following the scenarios, participants were interviewed individually by two researchers. Footage from participants’ video glasses was used to elicit the interviews in an attempt to explicate perceptual knowledge that usually remains tacit and objectivize the ‘pre-reflective and not immediately verbalizable’ [14].

Participant’s interviews were exploratory, deriving from what they were sharing in order to remain rooted in their experiences. Participants viewed their first person video footage, pausing the video and voicing aloud their experiences and actions at any time. Participants largely led the interview by pausing the video at regular stages and sharing their experiences. The interviewer was also allowed to pause the video and explore the participants experiences. As the interview proceeded, issues were probed in more depth by the interviewer and reference was made to the played-back video glasses footage. This stimulated both memory and reflection as it was (re)occurring, and allowed discussion of the embodied nature of the interactions among people and materials in the simulation. By using such a technique we aimed to provide a richer insight into participant’s sense-making processes as they voiced aloud their actions, including their non-verbal communication (such as head movement and the interaction with their surrounding environment and materials). Aside from using the video file footage, interviews were minimally structured to allow emerging themes to remain as true as possible to the participants’ experiences.

## Analysis

Various phases of qualitative data analysis were undertaken. In this article we focus on this secondary phase that we conducted that was influenced from concepts drawn from complexity theory. First, all personal perspective video footage of students’ engagement in the simulation activity and of their post-simulation interview was reviewed independently and then together by two researchers (GG and TF). Reviewing the video footage in combination with the interview transcripts helped to ensure that the analysis was firmly rooted in the participants’ experiences. The video footage also complemented the interpretation of the transcripts (for example the ability to detect non-verbal communication and environmental factors in the video footage that were not present in the interview transcripts). After the first independent viewings and readings, the researchers agreed on dimensions for further focus, selecting complexity concepts other writers have identified as particularly pertinent to professional practice, as mentioned earlier: *emergence, fluctuations, feedback loops, and socio-material relations.*

In the first phase we identified notable emergent patterns, fluctuations, feedback loops, and actors in the system that were evident from the students’ personal perspective. This process requires multiple viewings in order to become sensitized to details of materials, bodies, technologies and social dynamics in the scenario, and the changing nature of their relations. The scenario itself is nested within broader systems of which only traces can be discerned: the routines and materials of the ward, the curriculum and pedagogy underpinning the planned simulation, the medical and ethical protocols invoked in the practices that students were performing, etc. As a complex set of systems, emergence is continual. The viewer must decide which emergent patterns are most important to note. This may not be evident until the viewer establishes key occurrences in the video related to the students’ practice and learning, which requires a backtracking to follow webs of interactions that seem connected to such patterns. The process is repeated to identify ‘fluctuations’ and ‘feedback loops’, which are traced according to the emergent patterns selected for analysis. Different viewers may indeed select different patterns to trace, and also may be drawn to different social-material details to highlight. Note-taking requires thought and practice. One of us preferred to draw images and concept webs, while the other listed material interactions in a series of tables. We found that two researchers are invaluable for such work, especially when, as in our case, they bring two distinct backgrounds and ways of viewing – one of us a medical physician and the other an educational researcher. We also found that limiting the data (one 15-minute scenario video per student, in our case) was important to permit this in-depth analytic process which demanded so much re-viewing, pausing, back-checking and detailed discussion.

In the second phase, we analyzed each transcript interpretively, with constant reference to the video footage to examine: (1) students’ noticing and response to notable fluctuations; (2) students’ response to emergent patterns; (3) students’ strategies for managing materials; and (4) students’ response to different forms of feedback looping into the action. We shared analyses frequently to check our own predilections, omissions and emphases, then we conducted a comparative analysis across the students to identify key challenges, strategies, and implications.

## Results

Eight participants took part in the study with over 63 minutes of video footage and 311 minutes of interview data being captured. We will now consider participants’ challenges in dealing with complexity.

## Students’ challenges in coping with clinical complexity

The following challenges, derived from our analysis, particularly seemed to affect students’ ability to perform clinically as well as they felt they should have.

### Unprepared for ‘diving in’

Some participants described the ward experience as discomforting and felt they had not managed to figure out what was happening until too late, or to find some stability. Their video footage often panned in what seemed aimless sweeping.

#### Participant 1:

“It just felt like there was lots of things coming at me and there was loads of things to take in …”

#### Participant 1:

**“**like patients in beds and then the ward sister … and there was other staff … everyone seemed to be moving quite quickly and you didn’t really know where to look, it just felt like organized chaos, more so than what I would be used to. Whenever you’re in a ward as a medical student you’re sort of like a ghost nearly, you know, and you don’t really soak up the anxiety that you probably would if you were working.”

Others, we noticed, waited longer before ‘diving in’ to the tumult of dynamic uncertainty and simultaneous demands. They seemed to determine their focus, then took time to study particular materials, clinical details and conversations related to that focus.

#### Participant 4:

“I always find in a scenario like that your, your mind just goes on really rapidly at the start and then you almost have to focus in on what’s important because when you first go in you don’t really know where it’s going to lead and what’s going to turn out to be important so at the beginning I’m always trying to take everything in and then trying focus in afterwards.”

They asked more questions of the charge nurse to establish the situation and assigned task. In other words, they seemed to prepare for working in complexity by attuning and focusing – selectively orienting themselves to key social and material dynamics.

## Caught in an escalating system

An example of this was typified in one of the simulation scenarios, where the participant was asked to examine an elderly patient who had a reduced level of consciousness due to a terminal illness. The doctor was then confronted by the patient’s anxious adult ‘son’ who acts aggrieved about the do-not-resuscitate agreement. A defensive ‘daughter’ arrives, and escalating anger was set in motion amplified by feedback loops of blame and counter-blame.

### Participant 2:

“… I was just standing there and this conversation was going on between two people and I was kind of just a bystander and I was wondering, you know, should I say something, should I just let it go on, I was waiting until the point that really there would have been something for me to say.”

New perturbations were introduced by the patient chart that some participants refused to let the son read, sometimes holding it in front of them like a barricade. Some participants became entrapped in the confines of the patient’s cubicle area; bounded by the patient’s bed, clinical curtains and the relatives standing at the only exit point.

### Participant 5:

“… everyone was just ignoring the patient …they were arguing over the bedside. Over her actual bed, it was awkward …because whether the patient’s unconscious or not it’s still not really appropriate to be arguing about her on the middle of a ward. I should have taken them to a different side room …”

Overall, the system in this cubicle was self-organizing, and in some cases, the participant simply became swept up in the escalating dynamics.

### Participant 4:

“what was more going through my head … he was already so argumentative with his sister I guess I didn’t want to provoke that situation …”

The video footage seems to become an observer, as though the participant felt helpless to intervene effectively to defuse the emergence, reduce the complexity, or even to move the squabbling family members out of the patient’s cubicle.

### Participant 4:

“At this point I found it hard to even get a word in even to say something cos I wanted to say to him that, he was very much saying if anyone else had signed it he wouldn’t mind.”

## Bounded by the presence of a ‘patient’

A related challenge is dealing with the complex system set in motion through relations with a patient. Educators using complexity theory have shown how even a simple conversation is extraordinarily complex with simultaneous issues of intimacy, sense-making, unpredictability, the demand of the other’s gaze, and the mutual constitution that emerges [9, 10]. In one of our scenarios, the participant is supposed to analyze a patient’s urine sample, which is sitting on a clinical bench near to the patient. The patient, an elderly gent, engages the participant in continuous conversation and flirtation (due to a side effect of his neurological medication). Some participants appeared to become captured by the patient. Their video is continually drawn to the patient’s face, and they participate in a conversation that they seemed unable to divert.

### Participant 8:

“I suppose maybe you would try and keep a bit of distance because you didn’t want to encourage him em,… and it made it difficult to know what to say back to him in case he then thought that was another encouragement to keep going.”

“You can actually see, from where my glasses are, from where I’m looking, I’m not even looking at him, I don’t want to encourage him. That’s dawned on me as I’ve watched it. I think you, a lot of this you only really realize whenever you watch back, don’t you? Like I knew I was uncomfortable and I sort of knew I was laughing but you don’t really realize the extent to which you were laughing and you’re avoiding him, until you actually watch it.”

Meanwhile analysis of the patient’s urine sample, despite being a relatively simple procedure, was conducted poorly and sometimes with egregious errors.

### Participant 8:

“I was just so distracted … I was trying to block him out so I didn’t really see what was going on around me. So … it was obviously off-putting.”

In such cases the participant seemed, upon being confronted with simultaneous demands, to lack strategies for prioritizing, or even for reducing the ensuing complexity (such as, for instance, by taking the urine sample out of the patient’s cubicle to perform the test in a quiet place with concentration).

## Unable to assert boundaries of acceptable practice

In practice situations of dynamic complexity, boundaries of acceptable practice are tested in myriad ways. One of our scenarios deliberately introduced this. The participant, after examining an eccentric patient and en route to another task, is engaged in a quick side conversation by a nurse. She invites the doctor to join a social media group of clinicians who apparently are posting particularly amusing examples of this patient’s infelicities. All participants reported later that, while taken aback, they understood immediately the ethical problem. However, only a few seemed able to respond in any way that asserted their own boundaries of practice. Most seemed to prioritize the collegiality of the relationship with the nurse.

### Participant 1:

“Because she [the nurse] was being really nice, actually, and I thought I don’t want to lose a potential friend on the ward. It sounds really shallow.”

### Participant 6:

“Not to get on anyone’s wrong side, because if it go out I suppose that I tattled, I don’t know what the consequences would have been for all those nurses and then being despised for the next three months.”

Participants expressed a deep unease with the conflict that they were presented with; challenging this unprofessional practice was tempered by the impact this would have on their working relationship. None thought – then or later – of responding in any way to curtail the social media activity itself, either by stating its unacceptability or following up to stop the activity, although this would be rather a sophisticated expectation of a medical student.

## Discussion and Conclusion

This study has provided insight into some of the fine-grained nuances of medical students’ challenges in coping with complexity in a ward-based simulation exercise. It was evident that participants encountered many of the characteristics of complexity as they attempted to navigate the dynamic social, material, emotional and clinical landscapes of the simulation exercise. The veracity of such a clinical simulation was dependent on the multidisciplinary approach in its creation and by using ‘living’ patients.

Not surprisingly, participants encountered a number of challenges in this simulation environment – challenges that were vividly apparent in their personal video footage. Literally the moment participants passed from the quiet waiting hall into the busy ward, simultaneous issues emerged along with a myriad of busily moving people, objects and sounds. At this point the video frames often swung wildly and repeatedly from side to side, as though participants were trying to take in everything, make sense of the complexity, and perhaps decide what to focus on first. Even when the same scenario was run with the same patient and staff actors (but a different medical student), we observed clear cases of emergence and self-organization: myriad interactions, non-linear dynamics among heterogeneous elements, novel and unpredictable emergent patterns, feedback from various sources prompting disturbances and disordering the patterns, held in tension with self-organizing dynamics. Many of these challenges will not be new to experienced clinicians: they are commonplace occurrences for health professions within the dynamic complexity of everyday care in clinical environments [15–18]. The useful contribution of complexity theory is the language it provides to make these dynamics visible, showing how they move, how they can amplify and escalate problematic patterns, or how they can open interesting possibilities.

The important question is how to help students cope with this complexity. Linear problem-solving and protocols based on single cause-effect logic may be of limited use. As we saw in the urine sample analysis, even protocols that students can perform expertly can fall apart within the dynamics of complex systems. Students need to learn explicit strategies for complexity.

This initial exploration of complexity in simulation-based learning has signalled potential opportunities for pedagogies that incorporate complexity.

## Possible strategies for managing complexity

Based on our analysis, and framed in a clinical context, we propose some possible strategies for managing complexity.

### Taking time to size up the system

Participants did better who took some time to study the ward, make sense of it, locate key resources, and ask key questions before starting their tasks. They were also less distracted during the scenario by unrelated sounds and movements outside their focus of activity.

### Attuning to what emerges

At the same time, within their activity focus, participants performed better when they noticed small key fluctuations and multiple feedback loops that changed the emerging pattern of the system of interest – the patient’s condition, conversation, or patient-family or colleague relationships. These participants seemed able to anticipate surprising directions and be a little more prepared for them.

### Reducing complexity

As Osberg and Biesta have argued, complexity reduction is one way that effective professionals intervene to prevent escalation of problematic emergent patterns, without repressing the dynamic energy and possibility of a living system [19]. Experienced doctors, for instance, may build in moments of ‘pause’ to recalibrate or realign system dynamics. They may have strategies to step away from a patient, or to focus the patient on something besides distracting conversation. They move objects and people that are creating problems, position themselves to avoid being trapped in clinical spaces, or re-ground their focus ‘back to the patient’ when other systems escalate.

### Boundary practices

One strategy in complexity reduction is knowing how to create boundaries. Professionals introduce boundaries of language to separate tangled issues, or boundaries of space to calm escalating emotional dynamics. They reinforce boundaries of policy and routines to constrain complexity and limit burgeoning but potentially damaging possibilities. They also endeavour to be clear about boundaries establishing what they are responsible for, then limit their involvement outside these boundaries. Some may feel pressured to take on much more, but boundary practices can help a doctor establish their role, for instance, when confronted by quarrelling family members.

### Working with uncertainty

Educators writing about complexity often talk about the importance of students learning, ultimately, to tolerate and even to embrace the uncertainty of complexity [8, 9]. This may feel uncomfortable, especially for those accustomed to seek control and certainty in practice. However, educators suggest that students can practice ways of flowing with the energy of the system, and respond by using the very possibilities and energies that are continually thrown up by the system. This is not unlike the training that is familiar in counselling, crisis management and other situations of high uncertainty and complex dynamics.

We need to develop and explore improved ways of introducing complexity into simulation training such as nested systems, emergence, disturbance, (potential) feedback loops, diverse elements, many points of interaction, some decentred organization. Furthermore there is a need to develop strategies to manage the complexity of living simulation, what is effective in different contexts, and how these might be taught to senior students and transfer to real practice.

The study was unique on two counts. First, we applied complexity theory to qualitative data of medical education. While literature advocating complexity theory is growing in medical education, the available empirical research is yet sparse. Second, the study employed personal perspective video, using methods and rationale that will be explained further on, which is a powerful tool whose use is yet limited in medical research. However, the findings of the study have to be considered within its limitations. The findings may not be readily transferable to other medical schools. However, given the theoretical and epistemological orientation in this study, generalisability was not an objective. Moreover, this study was exploratory in nature, identifying potential ways of understanding student experience in ward work drawing from analytic tools offered by complexity theory. Further research needs to take place to build our knowledge and evidence in this important area.

Furthermore we do not claim that the genuinely emergent complexity involving real patients and diseases in living hospital wards is the same as ward simulation, which contains various controls, theatrical manufacture, and artifice. However, ward-based simulation can be designed in ways that will generate forms of emergence, self-organizing and nested systems that afford students an embodied and immersive experience of working in complex adaptive systems. Our explorations highlight some students’ lack of capability for recognizing and dealing with this complexity. We suggest that educators consider what strategies students might learn, and how they might learn these, to be more successful. We also argue for further research to develop and test these and other pedagogical approaches that might allow students to cope better with complexity in simulation and real clinical environments.
